# Tick-Borne Encephalitis Virus Infects Rat Astrocytes but Does Not Affect Their Viability

**DOI:** 10.1371/journal.pone.0086219

**Published:** 2014-01-20

**Authors:** Maja Potokar, Miša Korva, Jernej Jorgačevski, Tatjana Avšič-Županc, Robert Zorec

**Affiliations:** 1 Celica Biomedical Center, Ljubljana, Slovenia; 2 Laboratory of Neuroendocrinology – Molecular Cell Physiology, Institute of Pathophysiology, Faculty of Medicine, University of Ljubljana, Ljubljana, Slovenia; 3 Institute of Microbiology and Immunology, Faculty of Medicine, University of Ljubljana, Ljubljana, Slovenia; Ghent University, Belgium

## Abstract

Tick-borne encephalitis virus (TBEV) causes one of the most dangerous human neuroinfections in Europe and Asia. To infect neurons it must cross the blood-brain-barrier (BBB), and presumably also cells adjacent to the BBB, such as astrocytes, the most abundant glial cell type. However, the knowledge about the viral infection of glial cells is fragmental. Here we studied whether TBEV infects rat astrocytes. Rats belong to an animal group serving as a TBEV amplifying host. We employed high resolution quantitative fluorescence microscopy to investigate cell entry and cytoplasmic mobility of TBEV particles along with the effect on the cell cytoskeleton and cell survival. We report that infection of astrocytes with TBEV increases with time of exposure to TBEV and that with post-infection time TBEV particles gained higher mobility. After several days of infection actin cytoskeleton was affected, but cell survival was unchanged, indicating that rat astrocytes resist TBEV-mediated cell death, as reported for other mammalian cells. Therefore, astrocytes may present an important pool of dormant TBEV infections and a new target for therapeutic intervention.

## Introduction

Tick-borne encephalitis virus (TBEV) is medically important human pathogen that causes one of the most dangerous neuroinfections in humans in Europe and in Asia [1]–[3]. TBEV is a member of the genus *Flavivirus*, family *Flaviviridae* [4]. Mature virus particles are enclosed within lipidic envelope which carry attachment molecules (E protein) for the host cell receptors (heparan sulfate) [1], [5]–[7]. The first and the most important host cells infected by TBEV are likely epidermal Langerhans cells (i.e. dendritic cells; [8]) which transport the virus to the lymph nodes and initiate the spread of infection to lymphoid compartments [3]. In some vertebrate species the virus is neurovirulent and crosses the blood-brain barrier (BBB), which isolates and protects the brain tissue from fluctuations in nutrients, hormones, metabolites, and from the direct influences of many endo- or exogenous compounds circulating in the blood [3], [9]. The mechanism by which TBEV invades the central nervous system (CNS) is not clear, and may include cytokine-mediated entry, entry of TBEV-infected cells of the immune system and infection of endothelial cells [10], [11]. After entering the CNS, neurons are primary targets, but other brain cells may also be infected [3]. Such cells are astrocytes which are positioned between synapses and endothelial cells, playing a role in neurovascular coupling [12], [13]. The infection of astrocytes may have unfavourable consequences for brain functioning. In general, astrocytes have many leading roles in the brain, including integrating neuronal functions, neuronal support, and the maintenance of BBB inter-endothelial tight junctions in normal and pathologic conditions [14]–[26]. It has been shown recently that brain TBEV infection alters the permeability of the BBB in mice [11], and astrocytes may be implicated in this process, since these cells regulate blood flow in the brain [12], [13]. In addition, the effects of neurovirulence can be observed relatively rapidly, compared to the clinical course of the disease [3], [27], which likely indicates that a reservoir of TBEV may exist in the CNS.

The aim of this study was to test whether astrocytes, the most numerous glial brain cells [17], can be infected by TBEV and to measure cytoplasmic TBEV particle dynamics in the initial phases after the infection, along with the astrocyte viability. The susceptibility to TBEV infection would make astrocytes a potential TBEV reservoir. We used rat astrocytes as a model cell, since rodents of several species are known to be TBEV amplifying hosts and may maintain TBEV through latent persistent infections [3], [28].

The results show that TBEV infects rat astrocytes and that the infection of a single cell, which progresses in time-dependent phases, is associated with changes in actin cytoskeleton, but astrocyte viability is unaffected. We propose that astrocytes represent an important reservoir of TBEV brain infection, which makes these cells a new target for therapeutic intervention. Given their tight morphological association with blood vessels, infected astrocytes may possibly affect the BBB and neurons.

## Materials and Methods

### Ethics Statement

The care for experimental animals and the euthanization of animals was carried out in strict accordance with the following ethical codes and directives: The International Guiding Principles for Biomedical Research Involving Animals developed by the Council for International Organizations of Medical Sciences and the Directive on Conditions for Issue of License for Animal Experiments for Scientific Research Purposes (Official Gazette of the Republic of Slovenia 40/85, 22/87, 43/07). The protocol for the euthanization of the animals used in our study was approved by the Veterinary Administration of the Ministry for Agriculture and the Environment of the Republic of Slovenia (permit No: 34401-29/2009/2), issued on 22.4.2009. We have followed the rule of Three R’s to reduce the impact of research on animals.

### Cell Cultures

Astrocyte cultures were prepared from cortices of 3 days old Wistar rats as described [29]. Cells were maintained in high-glucose Dulbecco’s modified Eagle’s medium (Invitrogen, Life Technologies, Carlsbad, NM, USA) containing 10% fetal bovine serum (FBS), 1 mM pyruvate, 2 mM glutamine and 25 µg/ml penicillin/streptomycin at 37°C, 95% air/5% CO2. After reaching confluence, cells were manipulated as described [30]. Prior the experiments cells were sub-cultured onto Lab-Tek™ chambered coverglass (Thermo Scientific) or onto 22 mm-diameter poly-L-lysine-coated coverslips and used within 6 days after plating. Vero E6 cells were used for the preparation of virus stocks and as productively infected control cell type after infection with TBEV. Cells were maintained in Dulbecco’s MEM with high glucose (DMEM GlutaMAX™; Invitrogen) supplemented with 10% FBS. Cultures were incubated at 37°C/5% CO2.) Unless stated otherwise all chemicals for maintaining cell cultures and experimental procedures were obtained from Sigma-Aldrich (Diesenhofen, Germany).

### TBEV Labelling and Cytotoxicity Test

Viral strains and isolates of TBEV can be classified into three subtypes: the European, the Siberian and the Far Eastern [31]. In this study we used the European TBEV strain Ljubljana 1 [32]. TBEV was grown 7 days on Vero E6 cells. Supernatant was collected and centrifuged twice at 4°C (10 min at 3200 × *g* and 5 min at 20800 × *g* in Eppendorf 5804R centrifuge). Pellet was resuspended in astrocyte growth medium and labelled with different concentrations of fluorescent lipophilic Vybrant® DiD labelling solution (DiD, Molecular Probes, Invitrogen) in µM: 20, 50, 100 and 200. Labelling was performed for 2 h at 37°C (Eppendorf Thermomixer Compact, 500 rpm). Afterwards, the unbound dye was removed by buffer exchange into Hepes 145 buffer (50 mM Hepes, 145 mM NaCl, pH 7.4; [33] by using illustra NAP-5 columns with sephadex G-25 DNA grade (GE Healthcare)). Labelled virus (conc. 10^10^ copies per ml) was diluted in astrocyte growth medium, aliquoted and stored at −80°C. Astrocytes were infected with 10^3^–10^7^ TBEV.

Cytotoxicity of TBEV in astrocytes and Vero E6 cells was tested at various time intervals: 4 h, 18 h, 48 h, 3 days, 6 days, 10 days and 14 days p.i. with Countess™ Automated Cell Counter (Invitrogen) according to manufacturer’s instructions.

### TBEV RNA Concentration

The concentration of TBEV RNA was measured using one-step quantitative real time RT-PCR [34]. Vero E6 cells were infected with TBEV and the virus concentration was measured in the supernatant collected from Vero E6 cells 7 days post infection (p.i.) before and after labelling TBEV with fluorescent lipophilic Vybrant® DiD labelling solution. Further on, in the experiments, we have used the TBEV in concentration of 10^8^ copies/ml, labelled with 50 µM DiD.

### Imaging

Imaging of fixed and live cells was performed with an inverted confocal microscope (Zeiss LSM 510 META, Carl Zeiss) using oil-immersion objective 63×/NA 1.4. For excitation of DiD dye He/Ne laser was used (633 nm), the emission light was filtered with long pass filter, with the cut off below 650 nm. The conjugate Alexa Fluor 488 was excited by argon laser (488 nm) and the emission light was collected through the band pass filter (505 to 530 nm). The conjugate Alexa Fluor 546 was excited by He/Ne laser and the emission light was filtered with long pass filter 560 nm. To eliminate possible bleed-through, the green and red emission fluorescence was acquired sequentially. In live cells the mobility of vesicles that expressed DiD fluorescence of labelled TBEV was recorded. Time series images were recorded in 2 s intervals for 2 min of total recording time. Experiments were conducted at 37°C (Heatable universal mounting frame, Carl Zeiss).

### Immunocytochemistry

The cells were washed with the phosphate buffered saline (PBS), fixed in 2–4% formaldehyde (prepared from paraformaldehyde) in PBS for 5–15 min at room temperature (RT) and permeabilized with Triton X-100 for 10 min at RT. The non-specific background staining was reduced by incubating cells in blocking buffer, containing 3% bovine serum albumin (BSA) and 10% goat serum in PBS, at 37°C for 1 h. The cells were then stained with primary antibodies, diluted into 3% BSA in PBS and incubated at 37°C for 2 h or at 4°C overnight. When two primary antibodies (raised in different species) were used, the staining was done sequentially. Afterwards, the cells were rinsed in PBS and stained with secondary antibodies at 37°C for 45 min. At the end of the staining protocol the cells were mounted onto glass slides using Slowfade Gold antifade reagent (Molecular Probes, Invitrogen).

Primary antibodies used were: anti-E 1∶10 [35], anti-β-Actin 1∶200 (Abcam), anti-α-Tubulin 1∶100 (Sigma), anti-Clathrin light chain 1∶300 (Synaptic Systems), anti-EEA1 1:300 (BD Biosciences) and anti-LAMP1 1:300 (Abcam). Secondary antibodies were Alexa Fluor® 488 goat anti-mouse IgG and Alexa Fluor® 546 goat anti-rabbit IgG (Molecular Probes, Invitrogen).

### Analysis

The mobility of fluorescently labelled vesicles was analysed by ParticleTR software (Celica, Slovenia). To describe vesicle mobility the parameters were calculated as described [36], [37]: step length (displacement of a vesicle in the time interval of 2 s), track length (TL, the total length of the analysed vesicle pathway), velocity and maximal displacement (MD; [36]). Vesicle mobility was analysed in cells from three independent astrocyte cultures. The analysis of the vesicle mobility was performed for epochs of 30 s.

Vesicle size was analysed with ImageJ software (available at National Institute of Health, USA, http://rsbweb.nih.gov/ij/). Fluorescent particles above the threshold level 8 pixels^2^ (0.14×0.14 µm^2^) were determined as vesicles, corresponding to the vesicle size area above 0.1568 µm^2^ to cover a broad span of imaged vesicles with different fluorescence intensities at slightly different z-positions within optical slice, similarly as described [38]. The extent of the co-labelled vesicles by fluorescent probes was determined by manually counting the observed fluorescent probes in the green and red channels.

Statistical significance was determined with the Mann-Whitney Rank Sum test. Values presented on graphs are expressed as mean ± s.e.m.

## Results

### Identifying Initial Steps of TBEV Infection in Astrocytes

**Figure 1:**
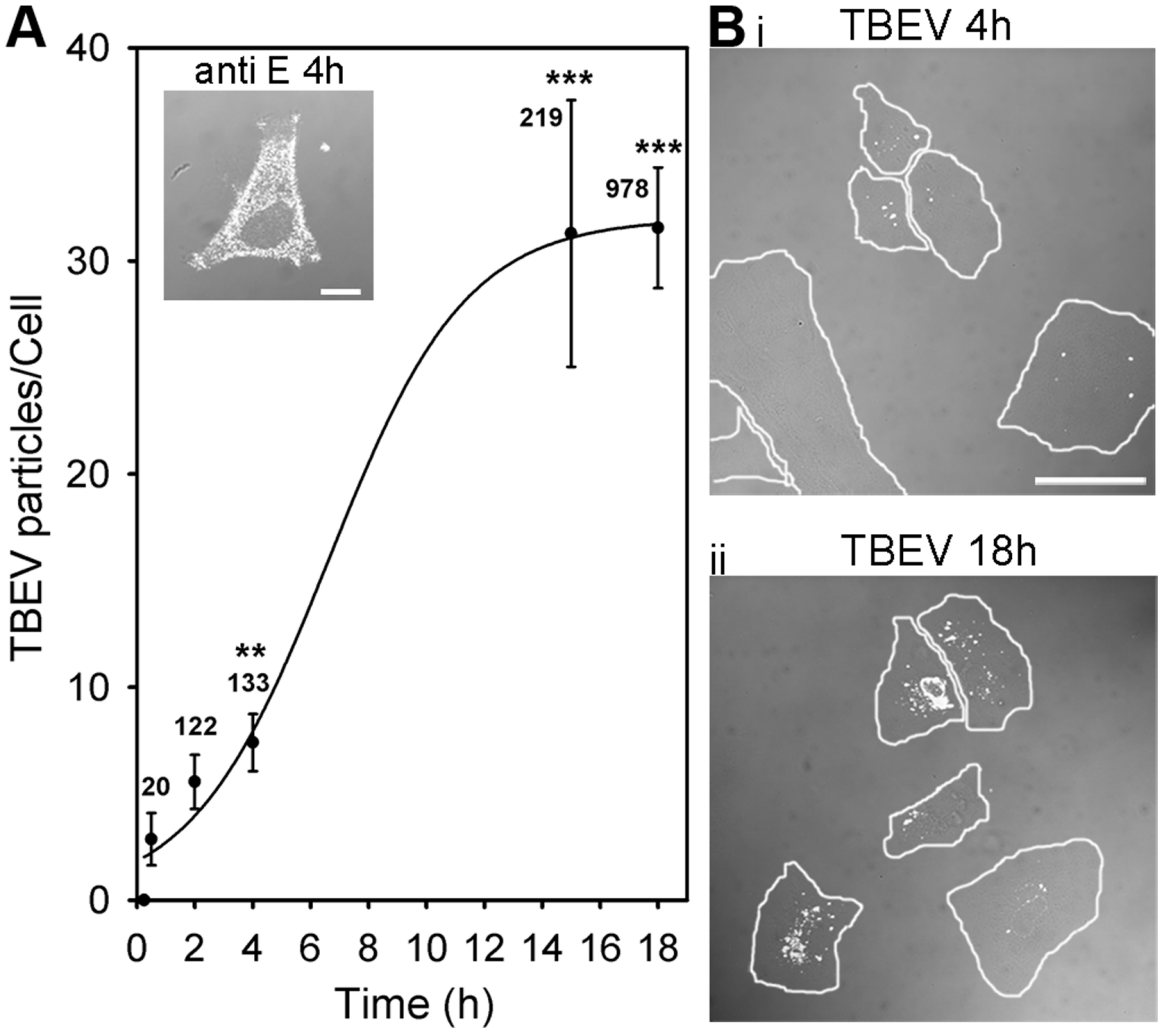
Time-dependant internalization of DiD-labelled TBEV into cultured rat astrocytes. (A) A DIC image at 4 h p.i. (inset) shows TBEV infection confirmed by anti-protein E labelling (anti-E 1∶10, white dots). Bar: 10 µm. The number of internalized DiD-TBEV particles per cell significantly increased with time p.i., from 2.8±1.2 particles/cell after 0.5 h to 31.5±2.8 particles/cell after 18 h, **P<0.01, ***P<0.001 *vs*. 0.5 h. Line represents sigmoid function of the form: f = a/(1+exp(-(x-x0)/b), fitted to the data. (Bi,ii) DIC microscope fields of DiD-labelled TBEV (white dots) infected live astrocytes after 4 h (i) and after 18 h (ii) p.i. Single cells are encircled. Bars: 50 µm. n = number of particles.

To image intracellular localization of TBEV particles, we pre-labelled them with 50 µM DiD lipophylic dye and afterwards infected cultured astrocytes with conc. of 10^7^ RNA copies/ml. Infection with TBEV was confirmed by immunolabelling with antibodies against the viral large envelope protein E [5] (Figure 1A, inset). TBEV labelling of cells increased with the time p.i. as assessed by counting TBEV particles per cell (Figure 1A,B). On one hand, the screening of several time periods following the exposure of cells to TBEV revealed that the number of TBEV particles per cell increased: 2.9±1.2 (0.5 h p.i.), 4.9±1.4 (2 h p.i., *P*≤0.05), 7.4±1.3 (4 h p.i., *P*<0.01) and 31.5±2.8 (18 h p.i., *P*<0.001) (Figure 1A). We analyzed up to 31 cells from three different animals. On the other hand, we also determined the fraction of cells in which at least 3 DiD-TBEV particles were observed in a coverslip with cultured astrocytes. Clearly, the percent of infected cells increased as a function of post-infection time: 68±9% (4 h), 92±2% (18 h; *P*<0.05; Figure 1B).

**Figure 2:**
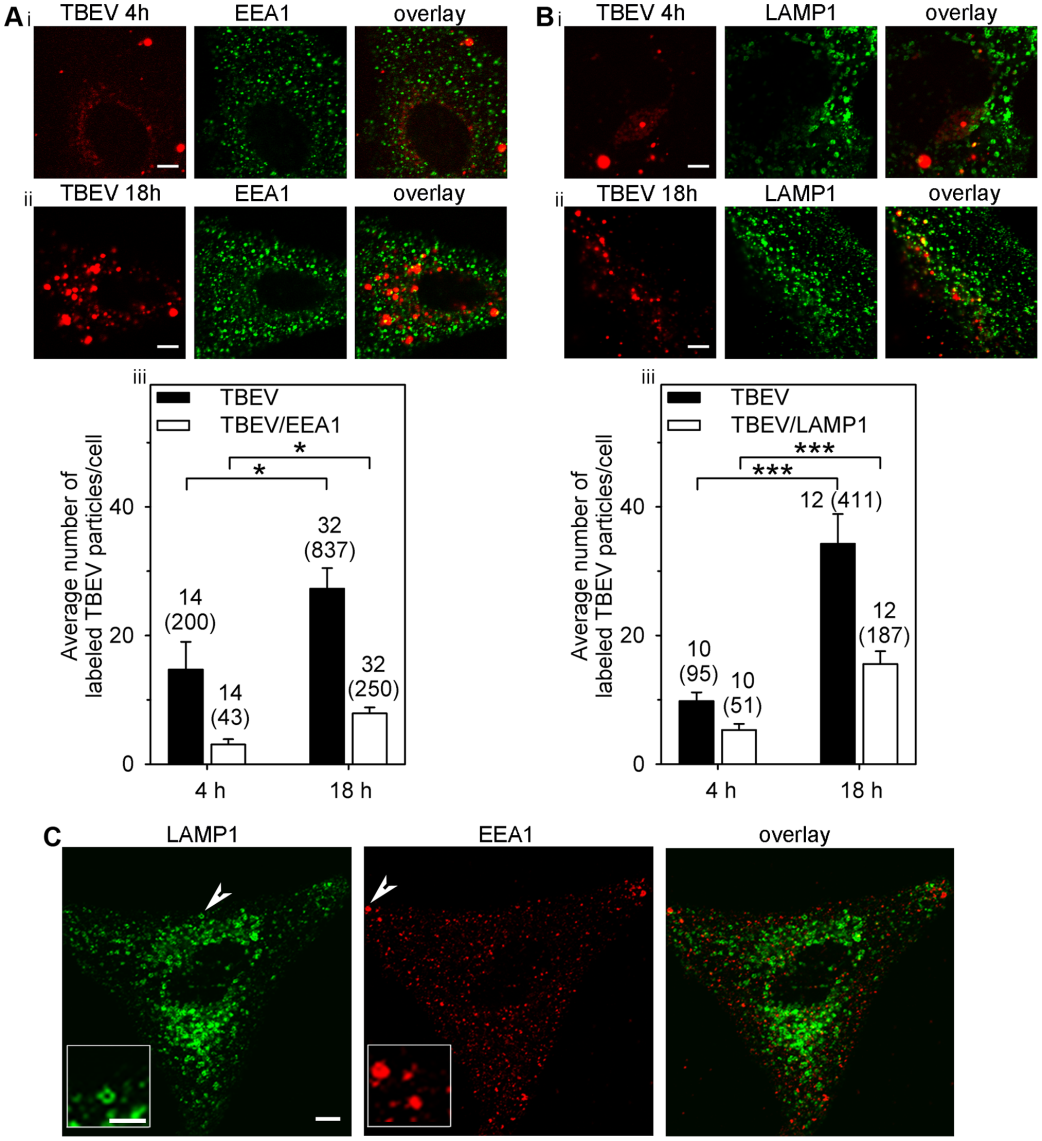
The number of endosomes and lysosomes associated with DiD-TBEV particles increased with time p.i.. (Ai,ii). An astrocyte with DiD-labelled TBEV vesicles (TBEV) incubated at 37°C for 4 h and 18 h and with labelled early endosomes (anti-EEA1 1:300). Overlays represent overlapped DiD-TBEV and EEA1 fluorescent signals, indicating the association between DiD-TBEV and endosomes. Bars: 5 µm. (Aiii). Prolonged incubation increased the average number of DiD-TBEV labelled vesicles per cell from 14.7±4.3 (4 h) to 27.3±3.2 (18 h) and also the average number of vesicles co-labelled with DiD-TBEV and EEA1 from 3.1±0.8 (4 h) to 7.9±0.9 (18 h). Black bars - DiD-TBEV labelled vesicles, white bars - DiD-TBEV and EEA1 co-labelled vesicles, *P<0.05. (Bi,ii) An astrocyte with DiD-labelled TBEV vesicles (TBEV) incubated at 37°C for 4 h and 18 h and with LAMP1-labelled late endosomes/lysosomes (LAMP1-lysosomal associated membrane protein 1; 1∶300). Overlays represent overlapped DiD-TBEV and LAMP1 fluorescent signals, indicating the association between DiD-TBEV and late endosomes/lysosomes. Bars: 5 µm. (Biii). Prolonged incubation increased the average number of DiD-TBEV labelled vesicles per cell from 9.8±1.4 (4 h) to 34.3±4.6 (18 h) and the average number of vesicles co-labelled with DiD-TBEV and LAMP1 from 5.3±1.0 (4 h) to 15.6±2.0 (18 h). Black bars - DiD-TBEV labelled vesicles, white bars - DiD-TBEV and LAMP1 co-labelled vesicles, ***P<0.001. (C) Astrocyte co-labelled with anti-LAMP1 (1∶300) and anti-EEA1 (1∶300). In a single, 1 µm thick optical slice, late endosomes/lysosomes and early endosomes appear to be largely different in size due to different position in z-axis and variable antibody attachments. Arrowheads point to large late endosomes/lysosomes (LAMP1) and early endosomes (EEA1). Bar (bar inset): 5 µm (2.5 µm). n (n) = number of cells (number of vesicles).

Next, to confirm that the localization of DiD-TBEV particles was intracellular, not merely at the cell surface, we immnuolabelled cells with antibodies against several proteins of the endosomal pathway. DiD-TBEV particles co-localized with clathrin light chain antibodies, 57±6% (n = 11) at 4 h p.i. and 48±6% (n = 16) at 18 h p.i. (*P*>0.1; data not shown), confirming clathrin-dependent endocytosis mediates TBEV entry. Additionally, DiD-TBEV particles were found co-localized with early endosomes (labelled with early endosomal antigen 1 (EEA1; [39]): 22% (at 4 h p.i.) and 30% (at 18 h p.i.; calculated from Figure 2Aiii) and with late endosomes/lysosomes (labelled with late endosomal/lysosomal associated membrane protein 1 (LAMP1; [40])): 54% (at 4 h p.i.) and 45% (at 18 h p.i.; calculated from Figure 2Biii). A few DiD-TBEV loaded vesicles appeared enlarged; they were comparable in size to late endosomes/lysosomes (LAMP1) and early endosomes (EEA1) in non-infected cells (Figure 2C). Taking into account that EEA1 and LAMP1 markers overlap in approx. 10% [41], we estimated that approximately 60–70% of DiD-TBEV particles were co-localized with either early endosomes or late endosomes/lysosomes and the rest were observed before entry into endosomal/lysosomal pathway, which is consistent with a multistep endocytic virus entry into animal cells [42].

### DiD-TBEV is Predominantly Loaded in Small Subcellular Structures

**Figure 3:**
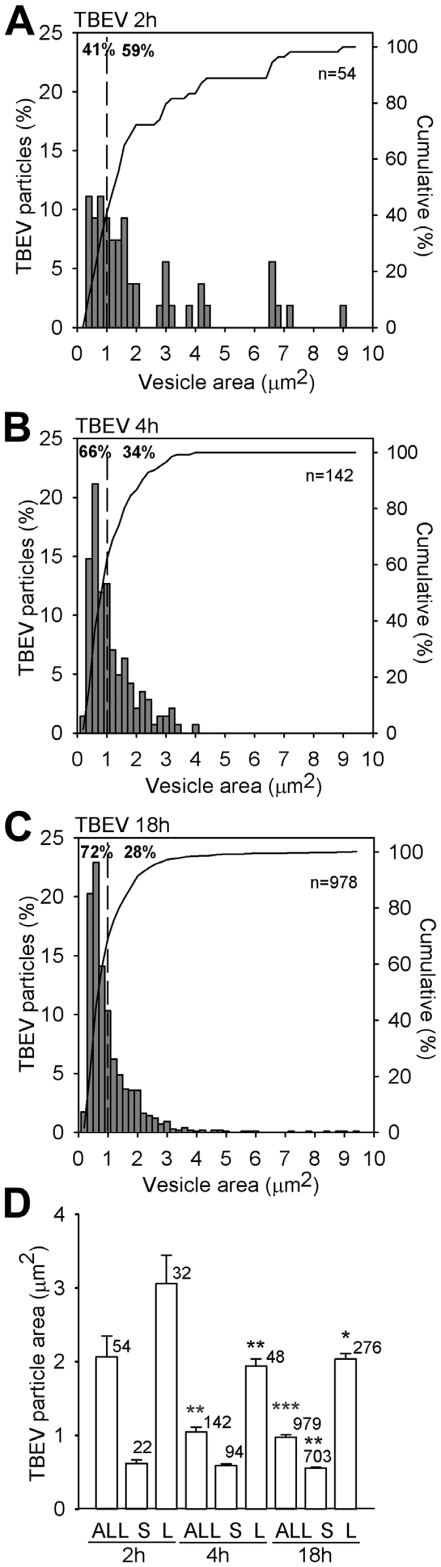
Changes in vesicle image area of DiD-labelled TBEV after internalization into astrocytes. Panels A–C show distributions of image areas of labelled DiD-TBEV vesicles. Note that the fraction (shown as %) of small TBEV vesicles, with image areas <1 µm^2^ (indicated by dashed lines), increased with the incubation time; from 41% after 2 h incubation (A), to 66% after 4 h incubation (B) and to 72% after 18 h incubation at 37°C (C). (D) The average vesicle area (in µm^2^) of TBEV vesicles significantly decreased with incubation time: 1.10±0.07 (4 h) and 0.97±0.03 (18 h) *vs.* 2.06±0.28 (2 h, **P<0.01, ***P<0.001). A similar decrease was observed in larger vesicles (L) with an image area >1 µm^2^ (in µm^2^): 1.94±0.09 (4 h; **P<0.01) and 2.03±0.08 (18 h) *vs*. 3.06±0.39 (2 h). The average area (in µm^2^) of small vesicles (S) remained roughly the same throughout all examined incubation times: 0.61±0.04 (2 h), 0.63±0.02 (4 h) and 0.56±0.01 (18 h; *P*>0.05). The significant difference was observed only between 4 and 18 h (**P<0.01).

We observed that internalized DiD-TBEV vesicles were predominantly small, as evident from vesicle area distributions (Figure 3A–C) and that their number increased with time p.i. (41% at 2 h, 66% at 4 h and 72% at 18 h). Vesicles with fluorescence area from 0.157 to 1.078 µm^2^ (55 px^2^) were described as small. Their apparent vesicle diameters estimated from fluorescence pixel area were between 440 and 1170 nm. However, the estimated diameters do not reflect the actual vesicle diameter due to the point spread function contributions to the image properties, which overestimates the actual vesicle diameters by more than three times [38]. Therefore, the diameters of smaller vesicles were likely between 150 and 400 nm. The average apparent vesicle area significantly decreased as a function of p.i. time. At 2 h p.i. the area was 2.06±0.28 µm^2^ (11 cells, 54 particles), at 4 h p.i. it was smaller: 1.10±0.07 µm^2^ (18 cells, 142 particles; *P*<0.01) and at 18 h p.i. it further reduced to 0.97±0.03 µm^2^ (31 cells, 979 particles; *P*<0.001) (Figure 3D). The observed decrease in vesicle image area can be assigned to a significant decrease in vesicle size of large vesicles (>1.078 µm^2^); their areas were reduced from 3.06±0.39 µm^2^ at 2 h p.i. to 1.94±0.09 µm^2^ at 4 h p.i. (*P*<0.05) and to 2.03±0.08 µm^2^ at 18 h p.i. (*P*<0.01) (Figure 3D).

### With Increasing p.i. time DiD-TBEV Particles Acquire Increased Directionality

**Figure 4:**
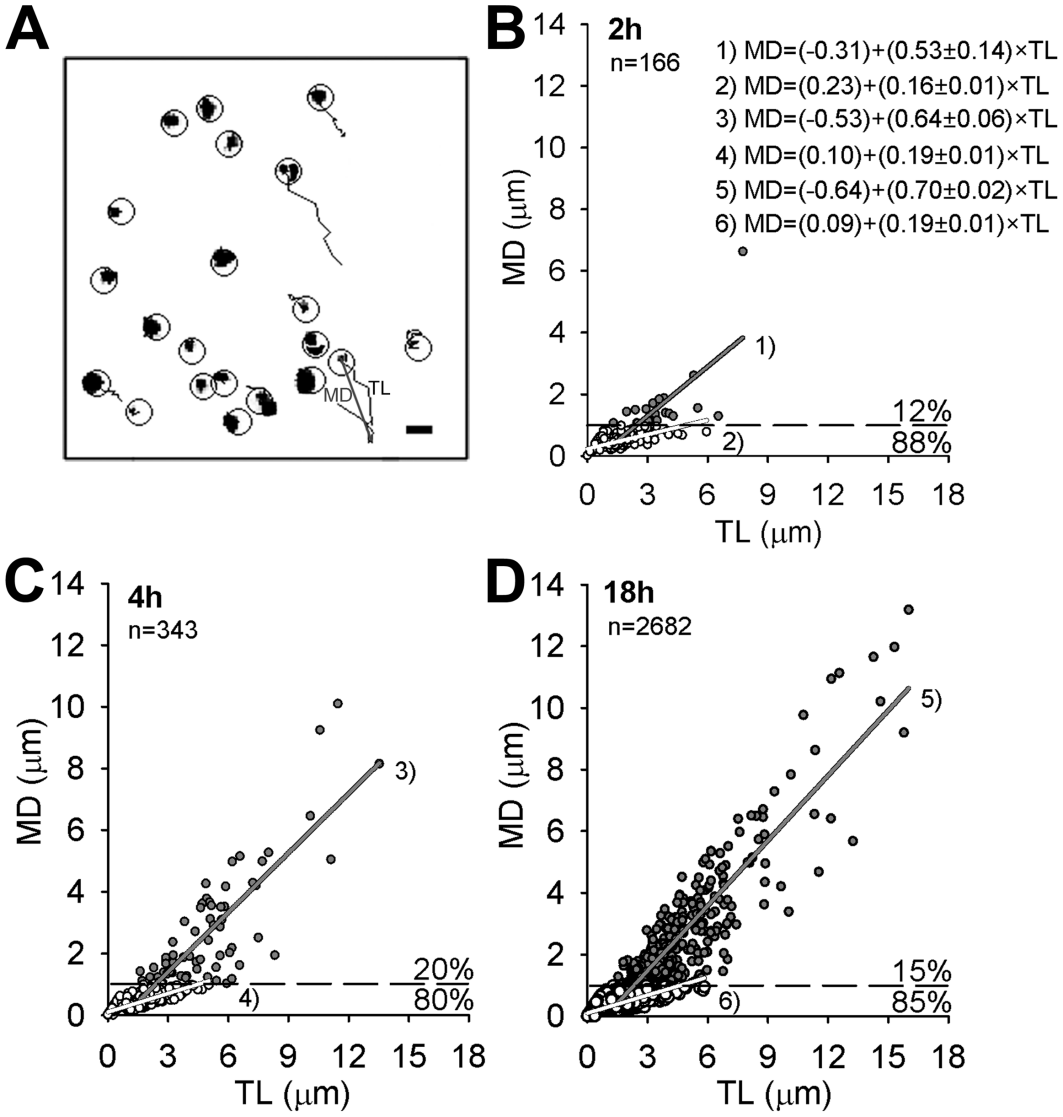
The percent of directional vesicle periods was less than 20%. Panel A shows vesicle paths in 30-directional mobility (NDM) and elongated trajectories represent directional mobility (DM). Circles denote vesicles subjected to mobility analysis. Bar: 2 µm. Graphs B–D represent vesicle directionality (relationship between MD and TL). The proportion of DM periods (MD>1 µm, dashed line) remained similar during all incubation times, although the number of vesicles significantly increased (see also Figure 1). White circles - NDM periods, grey circles - DM periods, n = number of vesicles. Values MD and TL were calculated at 30 s of recording time.

**Figure 5:**
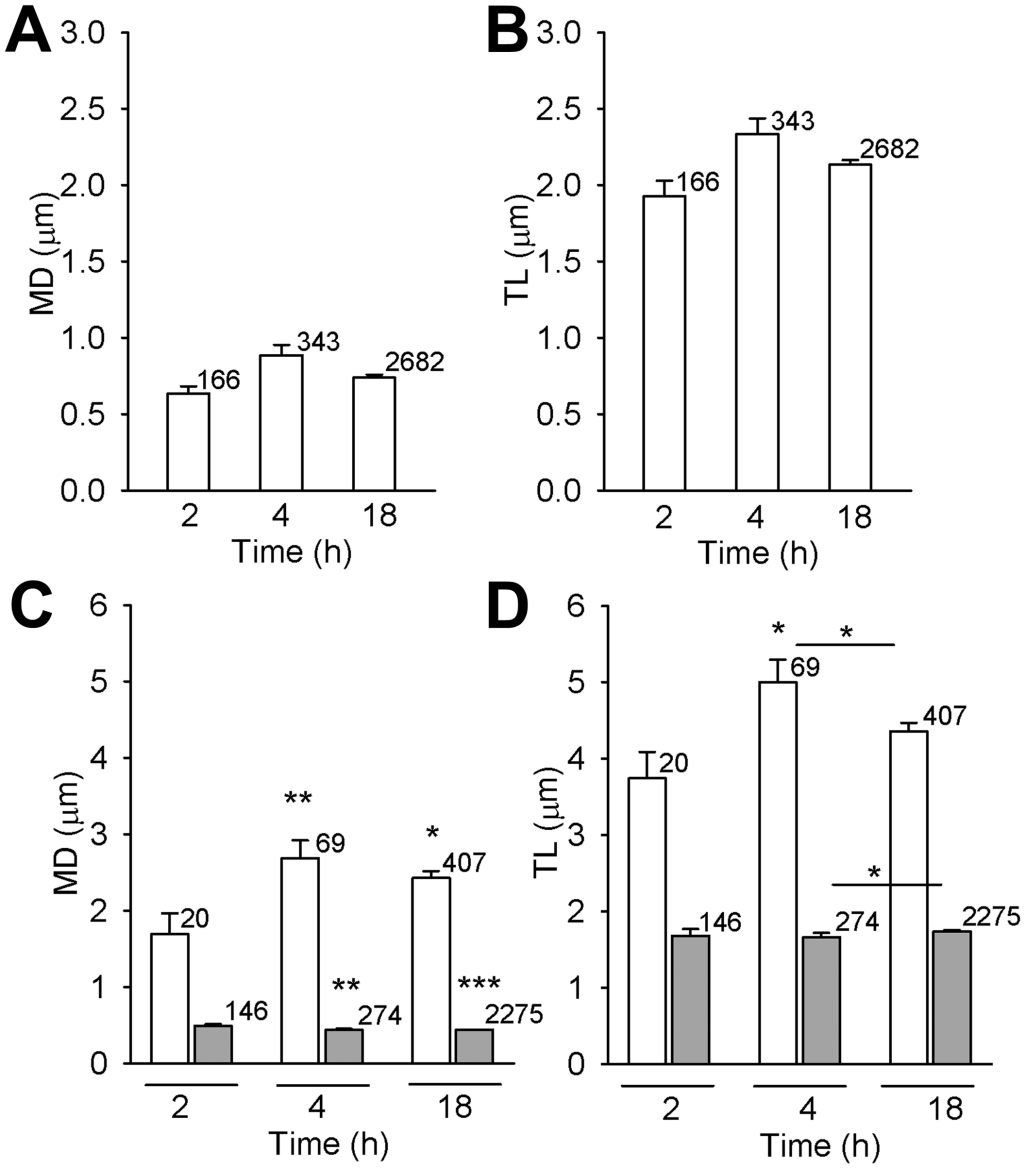
MD and TL of DiD-labelled TBEV vesicles exhibiting directional mobility increased with time p.i.. Average MD and TL of all vesicle periods are represented in panels A and B. No significant difference was observed neither between MD (in µm) at different times p.i.: 0.63±0.05 (2 h), 0.89±0.07 (4 h), 0.74±0.05 (18 h) (A), neither between TL (in µm): 1.93±0.10 (2 h), 2.23±0.10 (4 h), 2.13±0.03 (18 h) (B). However, a significant increase was observed for MD (in µm) for DM periods (white bars): from 1.69±0.27 (2 h) to 2.68±0.23 (4 h; **P<0.01) and 2.42±0.09 (18 h; *P<0.05) and small, but significant decrease for NDM periods: 0.48±0.02 µm (2 h), 0.43±0.01 µm (4 h) and 0.44±0.001 µm (18 h) (**P<0.01, ***P<0.001 (grey bars) (C). Mean TL (in µm) of DM periods (white bars) slightly, but significantly increased from 3.74±0.35 (2 h) to 5.00±0.29 (4 h; *P<0.05) while of NDM remained similar: 1.68±0.09 (2 h), 1.66±0.06 (4 h) and 1.74±0.02 (18 h) (D). n = number of vesicles.

Large vesicles with fluorescence area above 1.078 µm^2^ were immobile. On the other hand, small vesicles were mobile and were subjected to mobility analysis. Speed, track length (TL) and maximal displacement (MD) were calculated as described previously [36]. In 30 s of recording time vesicle pathways appeared predominantly clumped, meaning that vesicles exhibited non-directional mobility (NDM). However, some vesicles displayed directional mobility (DM), as evident from elongated trajectories (Figure 4A). The percent of DM periods (Figure 4, MD>1 µm, (dashed line)) remained below 20% during all incubation times, although the percent of small vesicles increased from 41% (2 h) to 72% (18 h, Figure 3). MD and TL of DM periods and NDM periods were distinct (Figure 5). Mean MD of DM periods significantly increased: from 1.69±0.27 µm (2 h), to 2.68±0.23 µm (4 h; *P*<0.05) and 2.42±0.09 µm (18 h; *P*<0.01) (Figure 5C). And mean TL of DM periods significantly increased from 3.74±0.35 µm (2 h) to 5.00±0.29 µm (4 h; *P*<0.05, Figure 5D) and to 4.36±0.11 µm (18 h). On the other hand, the mean MD and TL of NDM periods slightly decreased; MD: 0.48±0.02 µm (2 h) to 0.43±0.01 µm (4 h; *P*<0.01) and 0.44±0.001 µm (18 h; *P*<0.001) (Figure 5A), TL: 1.68±0.09 µm (2 h) to 1.66±0.06 µm (4 h; *P*<0.01) and 1.74±0.02 µm (Figure 5B). The average speed of DM periods were 0.12±0.01 µm/s (2 h) and 0.17±0.01 µm/s (4 h) µm/s and 0.15±0.003 µm/s (18 h) and of NDM periods 0.06±0.003 µm/s (2 h), 0.06±0.001 µm/s (4 h) and 0.06±0.01 µm/s (18 h).

### TBEV Induces Disintegration of Actin Filaments While Cell Viability Remains Unchanged

**Figure 6:**
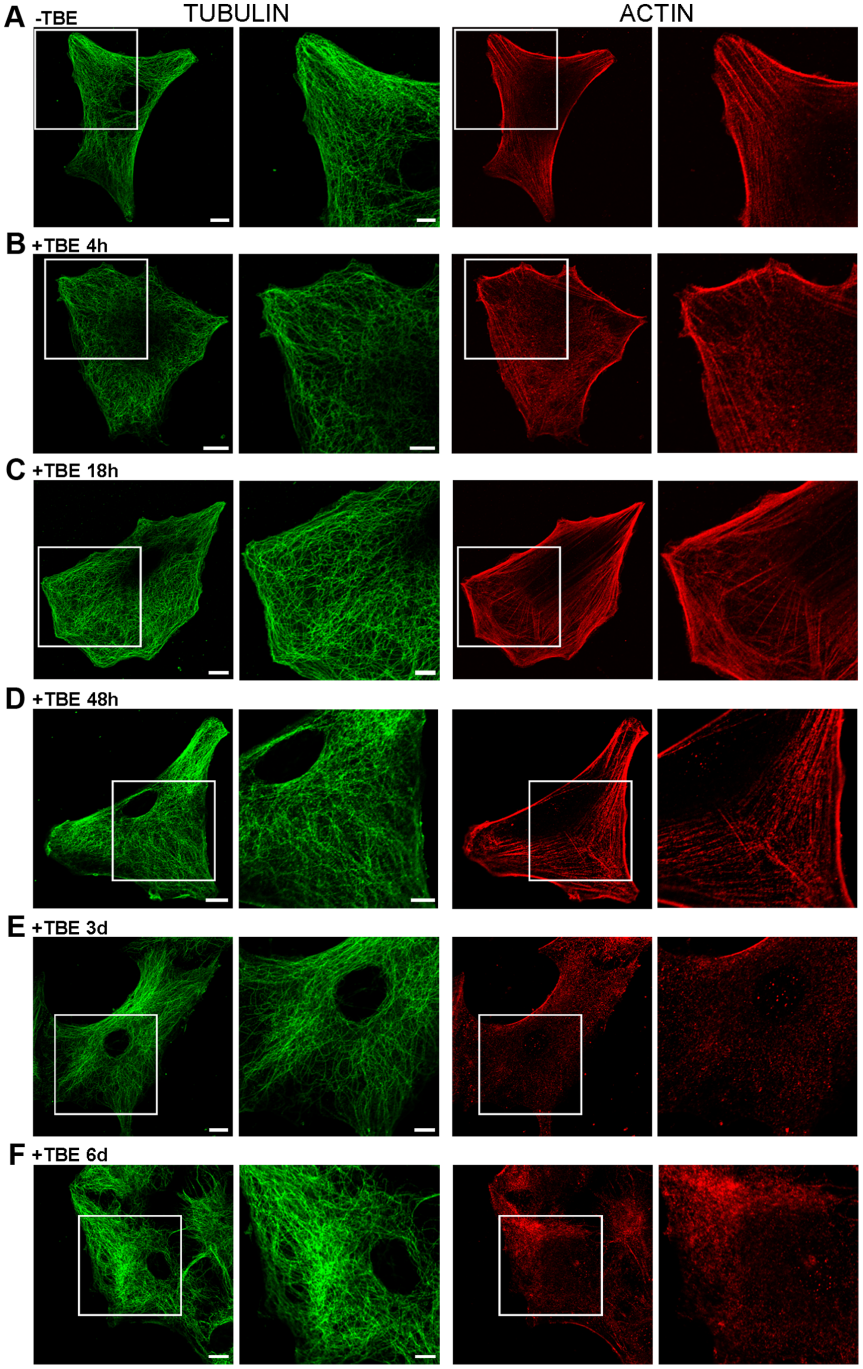
TBEV affected cytoskeleton morphology, but not cell shape nor cell viability. A–F, Immunocytochemical labellings of TUBULIN (anti-α-Tubulin 1∶100) and ACTIN cytoskeleton (anti-β-Actin 1∶200). Squared area is 2× enlarged in adjacent columns. Substantial rearrangement of actin cytoskeleton was observed after 3 and 6 days p.i. (E,F). Cell shape remained preserved at all times p.i. Bars: 10 µm (whole cell), 5 µm (enlarged panels).

Cytoskeleton rearrangements of host cells are reported for several viral infections (Herpesvirus, [43]; TBEV, [27]). To examine whether TBEV infection triggered changes of the rat astrocyte cytoskeleton, molecular motor-associated microtubules and actin filaments were labelled in astrocytes at several p.i. times (4 h, 18 h, 48 h, 3 days and 6 days). No obvious alterations of cytoskeleton were noticed in rat astrocytes until day 2 p.i. Then, after day 3 p.i. significant reorganization of actin filaments was observed, while microtubules appeared unaffected (Figure 6).

**Figure 7:**
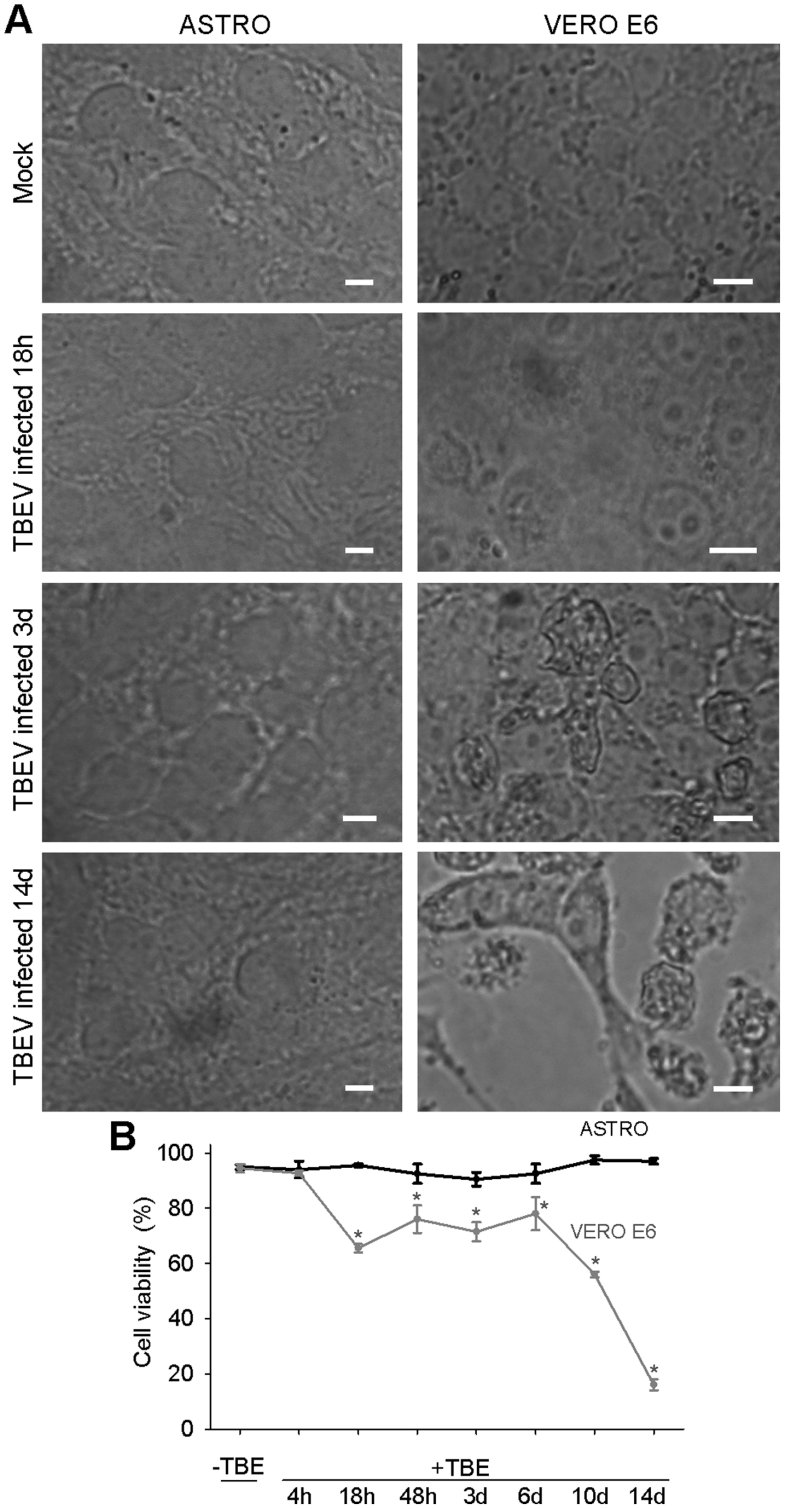
Prolonged infection with TBEV had no affect on the viability of rat astrocytes. A, Fields of view of non-infected (Mock) and TBEV infected primary rat astrocytes and Vero E6 cells recorded at different times p.i.: 4 h, 18 h, 48 h, 3 days (3d), 6 days (6d), 10 days (10d) and 14 days (14d). TBEV triggered CPE (morphologically altered cell shape, detached cells) only in Vero E6 cells, where the first CPE appeared at day 3 p.i. No CPE was observed in primary rat astrocytes. Bars: 20 µm. B, The viability of primary rat astrocytes was similar between mock treated (95±1%) and TBEV infected astrocytes at different times p.i.: 94±3% (4 h), 96±1% (18 h), 93±4% (48 h), 91±3% (3d), 93±4% (6d), 98±2% (10d), 97±1% (14d) (One Way ANOVA, P = 0.348), while in Vero E6 cells TBEV infection significantly reduced viability vs. non-infected cells (95±2%): 93±1% (4 h, P = 0.701), 66±2% (18 h, P<0.001), 76±5% (48 h, P = 0.004),72±3% (3d, P<0.001), 78±6% (6d, P = 0.008), 56±1% (10d, P<0.001), 16±2% (14d, P<0.001, One Way ANOVA). First significant diminishment in viability of Vero E6 cells was observed at 18 h p.i.

**Figure 8:**
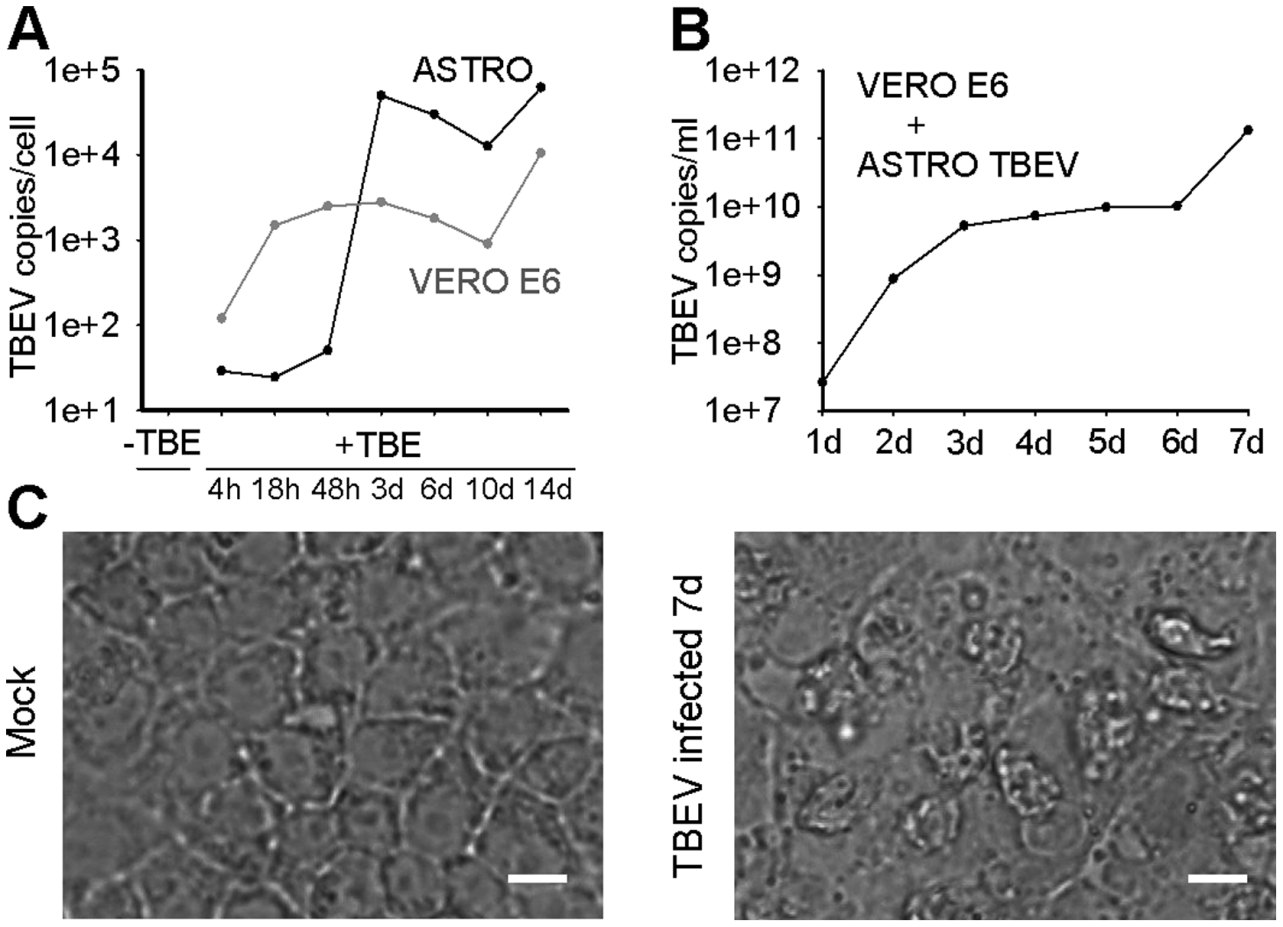
The infectivity of TBEV was preserved after long term growth in astrocytes. A, The graph represents the increase in the virus load (TBEV copies/ml) in primary rat astrocytes and in Vero E6 cells. In both cell types the virus successfully replicated. B, Vero E6 cells were infected with astrocyte supernatant (VERO E6+ ASTRO TBEV) collected at the end of the experiment and then the virus load was measured for 7 consecutive days in Vero E6 cells. The virus load increased to 1.32×10^11^ TBEV copies/ml and induced CPE in Vero E6 cells (C), confirming the infectivity of TBEV after long term growth in primary rat astrocytes. Bars: 20 µm.

Finally, to asses whether TBEV triggers the cytopathic effect (CPE) in primary rat astrocytes, we recorded fields of view of TBEV infected primary rat astrocytes and control Vero E6 cells and measured their viability for 14 days (Figure 7). The CPE (morphologically altered cell shape, detached cells) was observed in Vero E6 cells at day 3 p.i, whereas in astrocytes no CPE was recorded (Figure 7A). Morevover, the results of the trypan blue exclusion viability test show that the viability of primary rat astrocytes was not affected by TBEV infection (Figure 7 B). On the other hand, in the control Vero E6 cells, which were used to multiply TBEV, the viability was significantly affected already after 18 h (66±2%, P<0.001) and was further reduced to 16±2% (P<0.001) at day 14 p.i., compared to non-infected cells (95±2%) (Figure 7B). Successful replication of TBEV in both cell types was confirmed by measuring the virus load (TBEV copies/cell) at different times p.i. (Figure 8). Moreover, the supernatant collected from astrocytes at the end of the experiment (14 days) still reduced the viability of Vero E6 cells (39%) at day 7 p.i. and triggered CPE (Figure 8), confirming the presence of infective TBEV. From these data we conclude that TBEV infection does not significantly affect the viability of rat astrocytes, implying that these cells could act as a potential TBEV reservoir.

## Discussion

Despite of the high clinical importance of TBEV neuroinfection (long-term neurological disabilities, over 1% mortality rate, growing incidence in Europe), nothing is known about the TBEV infection of the most abundant neuronal supportive glial cells, astrocytes, and their possible implication in active or dormant TBEV infections. Recent data indicate that TBEV can be detected in rodent organs for longer periods of time and that the brain tissue stands out by high virus load [34]. In this report experimental evidences show that astrocytes, the most abundant mammalian glial cells become infected with TBEV, which makes them a potential mediator of brain infection and a reservoir of brain TBEV in rodents.

TBEV internalization into astrocytes increased with longer post-infection time. Although the mechanism of internalization remains to be elucidated, a number of different proteins have been identified as potential flavivirus receptors on the cell surface, however there is no direct evidence for a role of any of these during TBEV entry into host cells, and some of the results are controversial [44]. Glycosaminoglicans, such as heparan sulphate (HS), are expressed on different tissues [45]. Frequently, HS appears to serve as a primary attachment molecule that concentrates viral particles on the cell surface and may facilitate the subsequent binding to more specific receptor molecules [7], [46]. An involvement of HS during attachment and entry of TBEV has been demonstrated, although it appears that more than one single type of host-cell molecule is involved [3], [5], [7], [47]. In astrocytes mimicking injury response, upregulation of HS and higher sulfation of heparan sulphate proteoglycans occurs [48]. Therefore, one possible explanation of increased time-dependent TBEV entry into astrocytes could be attributed to an increased expression of HS on astrocyte membrane of infected astrocytes. However, this was not monitored in our experiments. The internalization of TBEV particles into astrocytes was consistent with confirmed clathrin-dependent entry of several members of *Flaviviridae* family: West Nile virus (WNV) [2], Dengue virus [49], [50], Hepatitis C virus [51] and Bovine Viral Diarrhoea virus (BVDV) [52]. And their localization in late endosomes/lysosomes was consistent with other flaviviruses: BVDV, [52]; WNV, [53]; DIL-labelled dengue virus, [50].

TBEV-loaded vesicles observed following 18 h post-infection time were predominantly smaller or comparable in size to early endosomes (300 nm, [2]; 300–400 nm, [54]); and much smaller from late endosomes which have on average 700 nm in diameter [55]. Taking into account the point-spread function related overestimation of vesicle diameter [38], the majority of TBEV vesicles exhibited diameters between 150–400 nm. This is in line with WNV vesicles, which have 100 nm in diameter until they start to fuse and become approximately the size of 500 nm [2]. A minor portion of DiD-TBEV particles (6% at 2 h p.i., 5% at 4 h p.i. and 20% at 18 h p.i.) was even comparable in apparent size to peptidergic and glutamatergic vesicles monitored in astrocytes (50–100 nm, [38]; [56]). The rest of TBEV-loaded vesicles were between 500 or 600 nm (diameter), which is in the range of late endosomes and lysosomes [54], [55]. With longer p.i. time the average size of large vesicles decreased, which indicates changes in vesicle dynamics, such as attenuated formation or enhanced cleavage of very large vesicles.

Despite the increase in the percent of smaller highly mobile TBEV loaded vesicles recorded with longer time p.i. (up to 72%), the percent of directional mobility periods remained below 20% at all times p.i. This is in line with the mobility properties of other vesicles which travel along the cytoskeleton [37], [57] and with the restricted availability of host cell trafficking apparatus to be used by internalized TBEV viruses (reviewed in Greber [58], [59]). These vesicles apparently move along both molecular motor-associated filaments. Analysis of vesicle speed revealed that the speed of DiD-TBEV vesicles generally corresponded to speeds of processive human myosins (Va, VI, and X) which travel as fast as 0.3–0.9 µm/s in vitro and 0.1–0.4 µm/s in live cells (references within [60]). These values correlate with DENV particle trafficking towards endosomes [61]. On the other hand, maximal recorded speeds above 1 µm/s confirm also the involvement of kinesin motors along microtubules [62]. Increased mobility at longer periods p.i. may reflect the changes in local protein synthesis affecting vesicle trafficking, as it was observed in axons infected by pseudorabies virus [63].

It is possible that viruses are transported on the account of innate cell vesicles, therefore we assessed if this impairs cell viability. In general, little is known about how various cell types in the CNS react to viral infections causing encephalitis [64]. In TBEV infected rat astrocytes we observed alterations of actin cytoskeleton, and no apparent change in the arrangement of microtubules. This is in contrast from the observation in human glioblastoma cells, where TBEV caused a substantial microtubule rearrangement already after 48 h p.i. [27]. The cell viability of primary rat astrocytes remained unaltered during monitored time p.i. (14 days). We did not observe any TBEV-triggered necrosis in rat astrocytes in 14 days p.i. (95.0±0.9% viable cells in the non-infected control and 97.0±1.0% viable cells after 14 days p.i., *P* = 0. 267). It appears that astrocytes are much more resilient to TBEV infection than the control Vero E6 cells (monkey kidney epithelial cell lineage) that showed reduced viability already after 18 h (Figure 7) and than porcine kidney cells, where almost all cells were dead at 50 h p.i. [65]. The shape of astrocytes was unaltered in 14 days p.i., the cells were not rounded, which is also in contrast to glioblastoma cell lines where 20% of cells were apoptotic already after 48 h p.i.; determined by rounding of cells and TUNEL assay [27]. These data suggest that in contrast to human glioblastoma cells rat astrocytes are more resilient to TBEV infection. Thus, rat astrocytes may serve as a reservoir for spreading the viral infection or maintaining TBEV reservoir in wild rodents. It has been reported that the virus likely remains dormant in the brain tissue for long time in wild rodents, while they show no clinical symptoms of TBEV infection [28], [34], [66]. We propose that the cell reservoir of dormant virus may consist of astrocytes. Vesicle traffic in astrocytes can be modified by therapeutics, such as fingolimod [67], which has been recently introduced to treat multiple sclerosis. Therefore, a similar strategy may be used to prevent astrocyte TBEV infection and possibly other brain cells. Recently, it was reported [11] that TBEV entry is independent on the breakdown of the BBB, but that the BBB breakdown is a consequence of TBEV brain infection. Whether astrocytes play a role in this process remains to be investigated, especially in the light of astrocyte role in compromising the permeability of the BBB.
